# Understanding the Unfamiliar: An Interpretative Phenomenological Analysis of Pediatric Residents’ Learning about Children with Severe Motor and Intellectual Disabilities

**DOI:** 10.31662/jmaj.2025-0504

**Published:** 2026-02-13

**Authors:** Manami Mizumoto, Junki Mizumoto, Hiroyuki Wakamoto, Mariko Eguchi

**Affiliations:** 1 Department of Pediatrics, Ehime Rehabilitation Center for Children, Ehime, Japan; 2 Department of Pediatrics, Ehime University Graduate School of Medicine, Ehime, Japan; 3 Department of Pediatrics, Ehime Seikyo Hospital, Ehime, Japan; 4 Department of Family Practice, Ehime Seikyo Hospital, Ehime, Japan; 5 Center for General Medicine Education, School of Medicine, Keio University, Tokyo, Japan

**Keywords:** interpretative phenomenological analysis, pediatrics, postgraduate education, severe motor and intellectual disability, situated learning

## Abstract

**Introduction:**

Children with severe motor and intellectual disabilities (SMIDs) require complex care involving both technical skill and emotional presence. Pediatric residents often lack meaningful exposure to such patients, and little is known about how they make sense of these learning experiences.

**Methods:**

We conducted a qualitative study using an interpretative phenomenological analysis to explore how pediatric residents experienced a three-month rotation at a facility for children with SMID in Japan. Four residents were interviewed twice during their rotations. Interviews were transcribed verbatim and analyzed through iterative coding and thematic development.

**Results:**

Three main themes emerged: (1) bewilderment at the unfamiliar and its overcoming, (2) confrontation with complex, ambiguous, and unstable medical conditions, and (3) psychological barriers to communication and their resolution. Initially, residents felt discomfort and emotional distance owing to unfamiliar devices, patients who were non-verbal, and the ambiguity of symptoms. However, through repeated contact, observational learning, and active participation in daily care, residents gradually developed intuitive judgment, comfort with uncertainty, and emotional connection. These experiences shifted their perceptions of children with SMID from passive and unknowable to responsive and relational. Residents also began to reconceptualize their role―not merely as problem-solvers but as care companions who tolerate ambiguity and foster connection.

**Conclusions:**

Pediatric residents initially struggled with unfamiliarity and uncertainty in caring for children with SMID. However, sustained exposure and interprofessional learning fostered emotional growth, intuitive competence, and epistemic humility. Training programs should provide longitudinal, hands-on experiences with patients with SMID and support reflective learning to cultivate more compassionate and capable pediatricians.

## Introduction

Pediatric residency training must equip physicians with the skills, knowledge, and sensitivity needed to care for a wide range of children, including those with severe motor and intellectual disabilities (SMIDs) ^(1), (2)^. Children with SMID often require long-term, multidisciplinary support owing to complex chronic conditions, frequent respiratory issues, and the need for assistive technology such as gastrostomy tubes, tracheostomies, and non-verbal communication aids ^(1), (3)^. Beyond the technical skills required for managing such cases, physicians must also develop capacities in patient-centered care, shared decision-making, and ethical reflection ^(3), (4)^.

Training residents for this complexity goes beyond technical instruction. It requires immersive clinical experiences that support both skill development and professional identity formation ^(5)^. However, many residents lack adequate exposure to children with developmental disabilities or medical complexity ^(6), (7)^, limiting their growth and potentially contributing to disparities in care ^(8)^.

Caring for children with SMID offers opportunities to engage deeply with essential themes in medicine: disability, caregiving, and human rights ^(9)^. Sustained relationships with patients and families can help residents better understand patient priorities, clarify their roles, and prepare for the challenges of complex care ^(4)^. However, little is known about residents’ learning process through their experience with such care. Exploring learners’ sense-making of ambiguity, vulnerability, or emotional connection in these contexts is essential to support their professional identity development. We hypothesized that residents’ experiences of care for children with SMID with their supervisors and other professionals may align with situated learning, or knowledge acquisition through active participation and social interaction within community of practice ^(10)^. That is, through daily care, residents will engage not just in clinical tasks but also in the emotional and ethical dimensions of caregiving, which help cultivate resilience, empathy, and a sense of belonging in a professional community.

This study explores pediatric residents’ learning processes of caring for children with SMID during a three-month clinical rotation at a specialized child development center in Japan. By focusing on this often-overlooked yet profoundly meaningful area of pediatric care, this study contributes to a deeper understanding of how clinical participation fosters professional growth in a clinical setting.

## Materials and Methods

### Interpretative phenomenological analysis

We used interpretative phenomenological analysis (IPA) to explore pediatric residents’ learning processes during their rotation at a center for children with SMID. IPA, rooted in Heideggerian philosophy, views individuals as shaped by their lived worlds and experiences ^(11)^. We acknowledge that we are influenced by the world we inhabit and the experiences we encounter, and through the interpretive processes between participants and researchers, we aimed to uncover ways residents made sense of their experiences, including meanings not immediately apparent to them. That is, IPA integrates phenomenological description with hermeneutic interpretation, allowing researchers to deeply engage with participants’ narratives ^(12), (13), (14), (15), (16)^. Through a double hermeneutic process, or the process by which researchers seek to understand participants who are attempting to understand their experience, we interpreted how residents themselves interpreted their experiences.

We chose IPA for its strength in evaluating emotionally charged, complex, and underexplored phenomena ^(12)^. This approach provided insight into ways residents engaged with disability, caregiving, and human relationships.

### Setting and participants

IPA emphasizes detailed evaluation of individual cases, using small, homogenous samples (4-6 participants) to enable in-depth understanding ^(12), (16), (17)^.

In Japan, postgraduate trainees complete a two-year general residency, followed by specialized training. This study occurred in a developmental and medical care center providing long-term residential care for children with SMID. Pediatric residents in postgraduate year (PGY)-4 or -5 rotate through the center for three months as part of their specialty training.

The center has 50 beds for children with conditions such as cerebral palsy, chromosomal and genetic abnormalities, metabolic disorders, in utero infections, abuse, and head trauma. Their ages range from infancy to older than 40 years. Children aged from 6 to 18 years attend a special support school connected to the facility.

During the rotation, residents manage 5-6 inpatients, conduct daily rounds, and provide care under supervision. Their tasks include medication and nutrition management, replacement of gastrostomy, nasogastric, and tracheostomy tubes, use of intrapulmonary percussive ventilation, and response to emergencies. They also work night shifts alone and participate in facility events, accompany patients to external appointments, and support outings. Residents also train in outpatient neurodevelopmental pediatrics.

### Data collection

We recruited all four pediatric residents who rotated at the center every three months over the course of one year. The period during which the study was conducted is omitted to protect the anonymity of the participants. The second author conducted in-depth interviews using a semi-structured interview guide (Appendix 1) twice in each three-month rotation: at two months and at the end. Interviews lasted 30-60 minutes. Only audio recordings were retained and transcribed. The interviewer allowed participants to guide the conversation while keeping focus on their lived experiences ^(12)^.

### Data analysis

We analyzed each case in depth, identifying themes, and then evaluated patterns across cases. IPA uses a stepwise, iterative process ^(12), (17)^:

(i) Reading and re-reading: Researchers immersed themselves in each transcript, reading it multiple times to deepen understanding.

(ii) Initial noting: Exploratory comments were recorded across three domains―descriptive (what was said), linguistic (how it was told), and conceptual (initial interpretations).

(iii) Developing emergent themes: Key segments were grouped into emergent themes that captured essential elements of the participant’s experience.

(iv) Searching for connections across emergent themes: Themes were evaluated for relationships and clustered into broader patterns.

We bracketed insights from one case before moving to the next to allow new interpretations. After analyzing all cases, we explored shared and divergent patterns, enriching interpretation. In the final stage, we applied relevant theory and presented findings as a narrative with quotes and commentary.

The first and second authors collaborated in the analysis, refining the codes through discussion. They shared the framework with the third and fourth authors for feedback. All four participants reviewed the findings to confirm resonance and offer corrections if needed.

### Reflexivity

Although seeking to understand participants’ worlds from within, IPA also draws on researchers’ perspectives to interpret meaning―this dual role is called the double hermeneutic ^(12), (16), (18)^.

The first author, a pediatric neurologist at the center and former resident herself, supervised participants and used both her empathy and clinical insight to guide interpretation. She aimed to represent participants’ voices faithfully while also using her own clinical and supervisory experience as interpretive resources. The second author, a health professions education researcher, had no prior relationship with participants and facilitated open dialogue. He adopted an inquisitive stance and helped anchor the study in IPA’s philosophical foundations. The third author, the center’s director and pediatric neurologist, reviewed findings with clinical insight. The fourth author, a pediatric educator overseeing the residency, contributed an educational perspective.

### Ethical considerations

The first author informed potential participants that participation was voluntary, with no negative consequences for declining. Written informed consent was obtained. Interviews were anonymized, and no incentives were provided. The Ethics Committee approved this study. We adhered to the Standards for Reporting Qualitative Research ^(19)^.

## Results

Four pediatric residents who participated in the rotation were included in the analysis. Three were in PGY-4, and one was in PGY-5. Three participants were younger than 30 years, and one was older than 30. All participants reported limited experience with caring for children with SMID in their two-year general residency.

We understand the importance of presenting detailed participant information so that readers can better interpret the findings and consider how these findings might apply to their own contexts. However, in this study, the number of participants was small, and all worked in a single, specific training program. Further details might inform colleagues or other insiders of individual identities. To safeguard participants’ anonymity, we refrain from reporting additional demographic characteristics.

Thematic analysis identified three main themes: (i) bewilderment at the unfamiliar and its overcoming, (ii) confrontation with complex, ambiguous, and unstable medical conditions, and (iii) psychological barriers to communication and their resolution. The following sections illustrate each theme using representative excerpts from participants’ narratives. We avoided gendered pronouns. Dialectal expressions were not translated. We tried to minimize the risk of identification of the participants when selecting, translating, and editing illustrative quotations. Quotations are listed in Table 1, Table 2 and Table 3. Table 4 illustrates the overview of these themes.

**Table 1.** Illustrative Quotes about Theme 1 “Bewilderment to the Unfamiliar and Its Overcoming.”

1-1	When I had to see them in the emergency room, how should I put it… it felt like there was this huge wall. As soon as a child came in, I’d think, ‘It’s a jūshin^†^ case. I have no idea what to do―this is too much for me.’ (†jūshin: an abbreviation of a Japanese term of children with SMID. )
1-2	Last year, a nurse asked me to change a tracheostomy cannula, and I just… I didn’t even want to touch it. I had no idea how to change it, and honestly, I just didn’t want to deal with it. […] The child had a tracheostomy, a gastrostomy tube, and just so many complications. I didn’t know where to start―it was like my mind was just overwhelmed and couldn’t make sense of anything.
1-3	The biggest thing was just how complex everything was. […] They were often on ventilators, and being tube-fed. On top of that, they were on so many medications. And sometimes, they’d have seizures too. I just felt completely overwhelmed―like I couldn’t handle any of it.
1-4	I had wanted to keep my distance from jūshin children―absolutely, I really did. Honestly, and maybe this is just my assumption, but I think most residents probably feel that way.
1-5	I had only just started learning pediatrics―on top of that, I had to take care of what I saw as an irregular child. It’s completely outside the scope of what I felt prepared for. It just felt like too much.
1-6	I’ve started to make sense of things, little by little.
1-7	I’ve had the chance to perform a lot of gastrostomy tube replacements, and I’ve also done some jejunostomy tube replacements. Last year, I almost never inserted nasogastric tubes, but now I’ve been placing them more regularly. So yes, I’ve learned a lot.
1-8	By gradually getting a sense of how things are done, the initial sense of discomfort or lack of confidence has been gradually fading. Even with something as seemingly simple as choosing a nutritional formula, thinking it through on my own gradually leads to a better understanding of what to do in different situations.
1-9	The sense of discomfort really faded a lot after working with the patients every day―handling the devices, managing the nutrition myself. I realized that even just seeing it makes a difference.
1-10	There weren’t that many children using special devices, so in that sense, it didn’t feel all that different from the acute care hospitals where I had trained before. I didn’t find myself feeling overwhelmed or taken aback very often.

**Table 2.** Illustrative Quotes about Theme 2 “Confrontation with Complex, Ambiguous, and Unstable Medical Condition.”

2-1	The patients already have some kind of underlying or chronic condition, and to make matters worse, there’s this acute change happening now―that part left me feeling a bit unsure, I guess.
2-2	Their background conditions are really complex, and compared to children without medical complexity, these children tend to deteriorate much more quickly once things start to go wrong.
2-3	Jūshin children are just different from typical children. Their symptoms often don’t show up clearly. For instance, I might draw blood and find an unusually high inflammatory response, even though there were few outward signs. It really makes me realize they’re not typical.
2-4	I picked up practical knowledge, like what to use when they’re prone to constipation. I feel like I’ve gained a lot of that kind of on-the-ground know-how.
2-5	When a child vomits or has some kind of trouble like that, I started to notice patterns―like maybe the feeding was stopped, or they were really tense. I began to get a sense of the kinds of situations where these things tend to happen.
2-6	Even if I don’t really understand their background or baseline, […] I feel like I’ve started to gain a bit of experience. Like, “that being said, this is abnormal”―I’ve kind of started to get that sense. […] I still don’t fully get what’s going on, but at least I can tell, “that being said, this is abnormal,” you know, when something just clearly feels off.
2-7	The nurses know much more than we do about the patients’ baseline vitals and their usual condition. They often tell me how the current situation differs from the child’s normal state.

**Table 3.** Illustrative Quotes about Theme 3 “Psychological Barriers to Communication and Their Resolution.”

3-1	What really shocked me when I first came was that few children could talk. It really hit me―like, I can’t have a conversation with any of them? [...] But now, little by little, I’ve started to be able to guess―like, maybe they’re feeling this or that―just by looking at their facial expressions.
3-2	I don’t know. I just saw them every day, and somehow… gradually, I guess.
3-3	What really struck me was how the nurses treated the children―with such affection. It was impressive. I thought, like, wow, that’s beautiful. I want to be like that too.
3-4	Honestly, I used to think these children wouldn’t really understand anything, no matter what we did, and that they wouldn’t respond. I had those prejudices. But now I get it―if I show them love and care, it really does come back to me. That’s something I’ve come to understand.
3-5	Last year, I saw some children, transferred from this center, at an emergency room. Their faces were so tense―they always looked painful. But now, at this center, their expressions are calm. That really made me think, yeah, this place is like their home. It’s where they live, where they feel comfortable. And it’s our job to protect that kind of space―that’s what our role is now.
3-6	I’d never really had any contact with people with disabilities in my life before, so… well, yeah… I mean, it sounds awful to say it like this, but I felt like I was not good at it. But as I spent time with more of the children, I started to feel this strong sense of affection growing inside me.
3-7	It’s completely different now. Back then, I honestly didn’t even know how to interact with them. I thought, like, “even if I talk to them, they probably won’t understand anyway.” But now, I can talk to them casually, play with them, and just hang out.
3-8	Everything I learned here was valuable. Including just getting used to being around Jūshin children. I really think it makes a big difference―whether we’ve had this experience or not. We don’t often get the chance to spend so much time focusing just on this kind of care, and in the future, So yeah, I definitely feel it’s been really beneficial.
3-9	I focused on how the other doctors do it. And, by observing how the nurses interact with the patients. I’d think, “Ah, maybe this is how I should do it.”

**Table 4.** Summary of the Main Themes.

1. Bewilderment at the Unfamiliar and Its Overcoming
**Initial bewilderment and distress**
• Limited prior experience, mostly in emergency/inpatient settings
• Discomfort when facing SMID cases in emergencies
• Main causes:
◦ Unfamiliarity with medical devices, medications, and nutritional management
◦ Perceived complexity of patients’ conditions
• Emotional responses:
◦ Anxiety about handling “irregular” cases
◦ Belief that such bewilderment is common among residents
**Process of overcoming bewilderment**
• Gradual acclimatization through repeated exposure
• Increased familiarity with care practices and devices
2. Confrontation with Complex, Ambiguous, and Unstable Medical Conditions
**Nature of conditions**
• Rapid deterioration and abrupt worsening without warning
• Symptoms often ambiguous and hard to interpret
**Impact on clinical decision-making**
• Uncertainty and confusion in identifying causes
• Differences from typical pediatric ward experiences
**Learning outcomes**
• Acquisition of practical, experience-based knowledge
• Importance of collaborating with nurses who know baseline patient conditions
3. Psychological Barriers to Communication and Their Resolution
**Initial challenges**
• Disorientation when verbal communication is hardly possible
• Uncertainty about understanding patient feelings
**Facilitating factors**
• Learning from nurses’ interactions
• Recognizing the center as a living environment
**Shift in perspective**
• From hesitation to affection and care
• Valuing this learning as a key takeaway from the rotation

SMID: severe motor and intellectual disability.

### Bewilderment at the unfamiliar and its overcoming. “All at Sea about Everything”

All participants reported that their prior experiences with children with SMID had been limited almost exclusively to emergency or inpatient care. In particular, the three participants in PGY-4 described that before they underwent the rotation at the center, they experienced significant distress (“this huge wall”) when required to see such patients in emergency settings. (Quote 1-1) As a reason for this strong puzzlement, the participant cited unfamiliarity with the use of medical devices, medications, and nutritional management specific to this patient population. This unfamiliarity became a primary source of their discomfort and avoidance (“I didn’t know where to start”). (Quote 1-2)

Another participant described in concrete terms how their bewilderment also stemmed from the complexity of the patients’ medical conditions. (Quote 1-3) This participant expressed the opinion that “most residents probably feel that way.” This statement reflected both an attempt to legitimize their own sense of bewilderment and an emerging awareness that this reaction may be a widespread issue. (Quote 1-4)

Another participant described these characteristics of children with SMID in emergency settings using the phrase “irregular child,” expressing their own anxiety in coping with such cases. This participant considered themselves a novice pediatrician with limited experience and thus emphasized the difficulty of managing an “irregular child.” (Quote 1-5)

All three PGY-4 participants reported that they overcame their initial sense of bewilderment through the rotation, when they saw numerous patients and gradually became more accustomed to the care. This acclimatization involved becoming familiar with medical devices and nutritional management, and acquiring various caregiving skills and techniques related to children with SMID. It also entailed a broader process of becoming personally comfortable with spending extended periods of time with these children. One participant reflected that gaining proficiency “little by little” in clinical procedures led to caring for children without hesitancy. (Quote 1-6, 1-7)

Another participant expressed the importance of accumulative experiences of caring for children with SMID because “even just seeing it makes a difference.” (Quote 1-8, 1-9)

The disappearance of bewilderment through familiarity was also supported by the account of a PGY-5 participant. This participant had already encountered many children with SMID during previous rotations at other institutions and had become accustomed to handling medical devices. Because of this prior experience, the participant reported feeling no sense of confusion or hesitation when caring for these children. (Quote 1-10)

### Confrontation with complex, ambiguous, and unstable medical conditions. “Stable or Serious?”

Participants recognized that when acute illnesses developed in hospitalized children with SMID, their symptoms were often difficult to interpret and could worsen rapidly. This led to uncertainty in clinical decision-making. One participant, reflecting on a case in which they struggled to treat worsening edema in a patient with cerebral palsy without being able to identify the underlying cause (“acute change happening now”), described their confusion as stemming from the differences between this setting and previous experiences on other pediatric wards. (Quote 2-1) The participant noted that the condition of these children deteriorates, and it tends to “deteriorate much more quickly” and without much warning. (Quote 2-2)

Another participant used the phrase “typical child” to contrast the distinctiveness of children with SMID. This reflected their perception of these patients as “not typical”. (Quote 2-3)

Although participants initially felt confused and uncertain, their experiences in the center enabled them to acquire “practical knowledge” about the signs and care approaches specific to children with SMID―knowledge that is not typically found in textbooks. Representative excerpts from their reflections are presented later. (Quote 2-4, 2-5)

Another participant reported developing an intuitive sense of “that being said, this is abnormal,” even when symptoms or signs were not clearly apparent in children. (Quote 2-6) This participant also recognized the need for specific strategies to manage such ambiguous situations. For example, relying on nurses who were well acquainted with the patient’s baseline condition proved to be particularly important. (Quote 2-7)

### Psychological barriers to communication and their resolution. “They Smiled Back at Me!”

One participant expressed a deep sense of confusion and disorientation when first encountering patients with whom verbal communication was not possible. However, through frequent contact with these patients, they gradually began to feel a sense of closeness. (Quote 3-1) When asked how they came to understand what the patients were feeling, the participant replied that they were not exactly sure. (Quote 3-2) As the interview progressed, the participant revealed that much of this understanding was influenced by observing how nurses interacted with the patients. (Quote 3-3, 3-4)

Participants recognized that the center was not merely a place for medical treatment but a place where children lived in a calm and comforting environment, “like their home”. They came to understand the importance of supporting this space. (Quote 3-5)

A participant reported a shift in perspective, from initial hesitation to developing a “strong sense of affection” and care toward the patients. (Quote 3-6) The participant replied that their feelings were “completely different” from before starting the rotation. (Quote 3-7)

Another participant also described a gradual dissolution of the psychological barriers they initially held toward children with SMID. The participant identified this process as one of the most significant learnings gained through the rotation, saying “I definitely feel it’s been really beneficial.” (Quote 3-8) This learning is promoted by the nurses and supervisors. (Quote 3-9)

## Discussion

This study explored how pediatric residents experience learning processes at a facility for children with SMID. Participants came to understand how to manage complex medical conditions and learned to communicate with children through repeated interactions with patients, devices, and professionals. This situated learning enabled them to cope with ambiguity and overcome emotional unfamiliarity.

Residents were initially overwhelmed by complex care, unfamiliar devices, and the absence of verbal communication. This discomfort did not reflect indifference but rather a struggle to interpret unfamiliar signs―prompting a recalibration of their clinical gaze. Health professionals often maintain psychological distance from patients they perceive as different or difficult to understand ^(20)^, a pattern also seen in psychiatry ^(21)^. Prior studies have shown that although most pediatric residents value the care of children with SMID, many feel frustrated by it ^(22)^. Our participants described children with SMID as “irregular” or spoke of “shutting everything out,” indicating an initial sense of otherness. However, through repeated care tasks and ongoing contact in legitimate peripheral participation, these reactions shifted to familiarity and affection. This transformation highlights the importance of sustained exposure in reducing bewilderment and fostering emotional connection as aligning with Allport’s contact hypothesis ^(23)^. The hypothesis insists that positive, direct interactions between members of different groups lead to more accurate perceptions and stronger intergroup affinity. It should be noted that this favorable learning circumstance is developed by ward nurses and supervisors. As one participant remarked, “even just seeing it makes a difference”―a phrase that underscores the effectiveness of being at the bedside in overcoming fear and stigma.

Residents came to address clinical uncertainty ^(24)^ by relying not just on protocols but also on experiential knowledge drawn from nurses and their own embodied sense-making. One participant’s comment―“That being said, this is abnormal”―captured this emerging intuition. Textbooks could not fully account for these nuanced judgments, and in the context of situated learning, an authentic activity promoted participants’ learning. Previous studies have shown that uncertainty complicates decision-making in complex pediatric care ^(25)^. Our findings suggest that learning to tolerate and navigate uncertainty, such as patients’ conditions and clinical courses, can be a critical skill. Developing comfort with ambiguity, supported by interprofessional collaboration, may promote more thoughtful and humane clinical decisions ^(26)^.

As residents adapted to this environment, they began to reconceive their roles―not as fixers applying algorithms, but as companions who offered presence and attention, such as affected contributors to a place where children lived in a calm and comforting environment. This shift reflects a form of epistemic humility ^(27)^ and relational growth essential for caring for children with profound needs. Recent discussions ^(27)^ suggest that epistemic humility is formed through relationships and is embedded within social contexts such as clinical encounters, and that its cultivation requires learning through practice and positive role models. The situated learning observed in this study may fulfill these prerequisites and thus foster participants’ epistemic humility.

Participants described transformative moments when they realized that children with SMID could respond and connect. These moments often arose by observing nurses’ interactions―such as a child returning a smile―revealing the child’s capacity for reciprocity. This finding highlighted the importance of community of practice as promotion for situated learning. These experiences reframed the children not as passive recipients but as relational individuals. Prior research shows that nurses consciously use their emotional responses to refine care ^(28)^. Residents observing the behavior of nurses indicated the value of interprofessional education. Although health care workers generally recognize the importance of communication with children with disabilities, barriers such as time constraints and task-oriented routines persist ^(29)^. Our findings suggest that modeling nonverbal communication and relational care, based on interprofessional education, could enhance residents’ communication competencies.

### Limitation

This study has several limitations. First, participants might censor their responses, opting for socially desirable answers ^(30)^. To minimize this, a non-supervisory interviewer conducted the interviews in a setting conducive to honest dialogue. Transcripts were anonymized before sharing with co-authors. In addition, interviewing each participant twice according to the progress of each rotation may contribute to efficient extraction of participants’ learning process. However, the small number of participants made complete anonymity difficult. We explained this limitation to participants in advance during informed consent. Second, our analysis reflects only the perspectives of faculty physicians; we did not include the voices of patients, families, residents, or other professionals. This may have constrained the interpretive range. Third, to protect participant anonymity, we omitted potentially identifying demographic and professional details. This was essential given the small, specific population, in which even aggregated data could risk identification. We recognize that this absence may limit readers’ ability to fully contextualize participants’ perspectives. We compensated for this absence by offering rich, thick descriptions supported by extensive verbatim quotations. By presenting participants’ words in detail and embedding them in the thematic analysis, we aimed to convey the depth and diversity of their experiences, enabling readers to interpret the findings with as much contextual richness as possible despite the necessary omissions. Such thick descriptions may contribute to the transferability of these findings. Considering many children resident at home or in long-term nursing care facilities, the findings may be interpreted even outside the context of hospital wards with children hospitalized long term. In such contexts, relationships with family and other professionals should be underlined.

The findings come from a small group of residents in a specific setting, and different environments may yield different experiences. Nonetheless, we grounded our analysis in the double hermeneutic of IPA―integrating participants’ meaning-making with our own interpretive lens. We deliberately used our positionality to stay engaged while avoiding over-identification, aiming for a reflexive stance that deepened analysis and strengthened interpretive credibility.

### Conclusion

This study illustrated how pediatric residents experienced bewilderment toward children with SMID, and how that discomfort gradually transformed into attachment and interest through everyday interactions. Residents learned to build relationships with such children while embracing ambiguity and difference, rather than trying to resolve or eliminate them. To foster such attitudinal change and revised perspectives on disability, training programs should ensure that residents spend ample time with patients, become familiar with various care practices, including device manipulations, and engage in rich learning opportunities from interprofessional collaboration.

## Article Information

### Acknowledgments

The authors thank all participants for sharing their experiences.

### Author Contributions

Manami Mizumoto and Junki Mizumoto contributed equally to this work. Conceptualization, methods, data curation, investigation, formal analysis, and writing―original draft were undertaken by Manami Mizumoto and Junki Mizumoto. Validation was performed by Manami Mizumoto, Junki Mizumoto, Hiroyuki Wakamoto, and Mariko Eguchi. Supervision was undertaken by Hiroyuki Wakamoto and Mariko Eguchi. Junki Mizumoto undertook visualization. Manami Mizumoto was responsible for project administration and resources. Writing―review and editing was undertaken by Hiroyuki Wakamoto and Mariko Eguchi. Manami Mizumoto and Mariko Eguchi share the role of the corresponding author.

### Conflicts of Interest

None

### Ethical Considerations

The Ethics Committee of the Ehime Rehabilitation Center for Children approved this study (reference number: 54).

## Supplement

Supplementary Material
