# Supplementary material for: Understanding the Unfamiliar: An Interpretative Phenomenological Analysis of Pediatric Residents’ Learning about Children with Severe Motor and Intellectual Disabilities
Source: JMA J. 2026 Feb 13;9(2):547–55. doi: 10.31662/jmaj.2025-0504 (PMC13058700; doi:10.31662/jmaj.2025-0504)
Supplement: Supplementary Material [file 2433-3298-9-2_0547-s001.pdf]

Appendix 1. Interview guide

What kind of pediatric residency training have you completed so far?
What has been the most memorable aspect of your training at the center? Why?
Will the training at this center be useful for your future career as a pediatrician? Why?
(Starting with the second interview, the following questions were added.)
Did you have any concerns when starting training at the center?
Did you encounter any confusion during your training at the center? Was it resolved?
How do you interact with hospitalized patients?
